# City-scale assessment of long-term air quality impacts on the respiratory and cardiovascular health

**DOI:** 10.3389/fpubh.2022.1006536

**Published:** 2022-11-10

**Authors:** Libor Šulc, Petr Gregor, Jiří Kalina, Ondřej Mikeš, Tomáš Janoš, Pavel Čupr

**Affiliations:** RECETOX, Faculty of Science, Masaryk University, Brno, Czechia

**Keywords:** air pollution, long-term data, respiratory health, cardiovascular hospitalizations, benzene, PM, urban exposome, high-resolution

## Abstract

**Background:**

The impact of the urban environment on human health is a contemporary subject of environmental research. Air pollution is often considered a leading environmental driver. However, a plethora of other factors within the urban exposome may be involved. At the same time, the resolution of spatial data is also an important facet to consider. Generally, systematic tools for accurate health risk assessment in the urban environment are missing or are not implemented.

**Methods:**

The long-term impact of air quality (PM_10_, PM_2.5_, NO_2_, benzene, and SO_2_) on respiratory and cardiovascular health was assessed with a log-linear model. We used the most accurate health data in high city scale spatial resolution over the period 2010 to 2018. Selected external exposome parameters were also included in the analysis.

**Results:**

Statistically significant associations between air pollution and the health of the urban population were found. The strongest association was between benzene and the incidence of bronchitis in the adult population [RR 1.552 95% CI (1.415–1.704) per 0.5 μg/m^3^ change in benzene concentration]. A similar relation was observed between NO_2_ and the same health condition [RR 1.483 95% CI (1.227–1.792) per 8.9 μg/m^3^ of change in NO_2_]. Other weaker associations were also found between asthma in children and PMs, NO_2_, or benzene. Cardiovascular-related hospitalizations in the general population were linked with NO_2_ [RR 1.218 95% CI (1.119–1.325) per 9.7 μg/m^3^ change in NO_2_]. The remaining pollutants were slightly less but still significantly associated with cardiovascular-related hospitalizations.

**Conclusion:**

Our findings are mostly highly statistically significant (*p* ≤ 0.001) and are in line with current literature on the adverse effects of air pollution on the human population. The results highlight the need for continual improvements in air quality. We propose the implementation of this approach as a systematic tool for the investigation of possible health risks over a long period of time. However, further research involving other variables is an essential step toward understanding the complex urban exposome and its implications for human health. An increase in data spatial resolution is especially important in this respect as well as for improving city health risk management.

## Introduction

Air quality (AQ) is becoming generally recognized as a significant factor affecting the health of the human population. According to the World Health Organization (WHO), poor ambient AQ caused three million premature deaths globally in 2012, of which Europe bears 479,000 deaths, and the Czech Republic bears 6,010 deaths (1). Particulate matter (PM_10_, PM_2.5_) and NO_2_ are examples of pollutants often found in high levels in urban areas (2, 3). Exposure to such pollutants may contribute to the promotion or development of various types of health issues. These can include, for example, lung cancer, cardiovascular diseases (CVD), chronic obstructive pulmonary disease, asthma, bronchitis, and pneumonia. The extent to which these diseases are caused directly by AQ has not yet been fully described. Nonetheless several studies have previously outlined a possible link between AQ and various aspects of human health (4–8).

The impact of environmental factors on the respiratory and cardiovascular systems has been reported in many scientific publications (9). Several of them worked with ecological study designs (10, 11). The ecological study is often used on city-level populations (12–14), on multi-city populations (15, 16), or on the national level (17). In this study, we used an ecological design but also aimed to increase the resolution of our data as much as possible. For this reason, we worked with subunits - ZIP areas (ZAs) in the city of Brno (Czech Republic) – similarly to Zhang et al. (18) with respect to the Yinzhou district, Ningbo, China. Regarding the temporal aspect, generally, the data window varies, but aggregation often focuses on short-term exposure (13, 14, 19, 20). Annual data focusing more on long-term exposure (17, 21) are also common. Our approach was geared more toward long-term exposure as we utilized annual, and 5-year means over 9 years.

However, AQ is not the only external factor contributing to the deterioration of human health. Other important factors include, among others, increasing temperature, the availability and quality of health care, and noise pollution. These and many more variables are relevant to the concept of the urban exposome. The urban exposome holistically considers all factors from conception till death and offers a more complex explanation of the connection between human health and socioeconomic/environmental pressures (22–24).

The objective of this study was to (i) describe the development of AQ and respiratory and cardiovascular health in the city of Brno over the 2010 to 2018, and (ii) assess the potential impact of selected AQ indicators on the respiratory and cardiovascular health of the population in the city of Brno, while considering other factors in the urban exposome with possible effects on human health. Emphasis was placed on the use of data with as high a resolution as possible to reveal long-term associations. These results may be further used by the city authorities in drawing up health risk management plans and policies. And approach used in this article may be also implemented as smart tool for systemic health risk assessment in the urban environment.

## Materials and methods

### Study area

**Figure 1:**
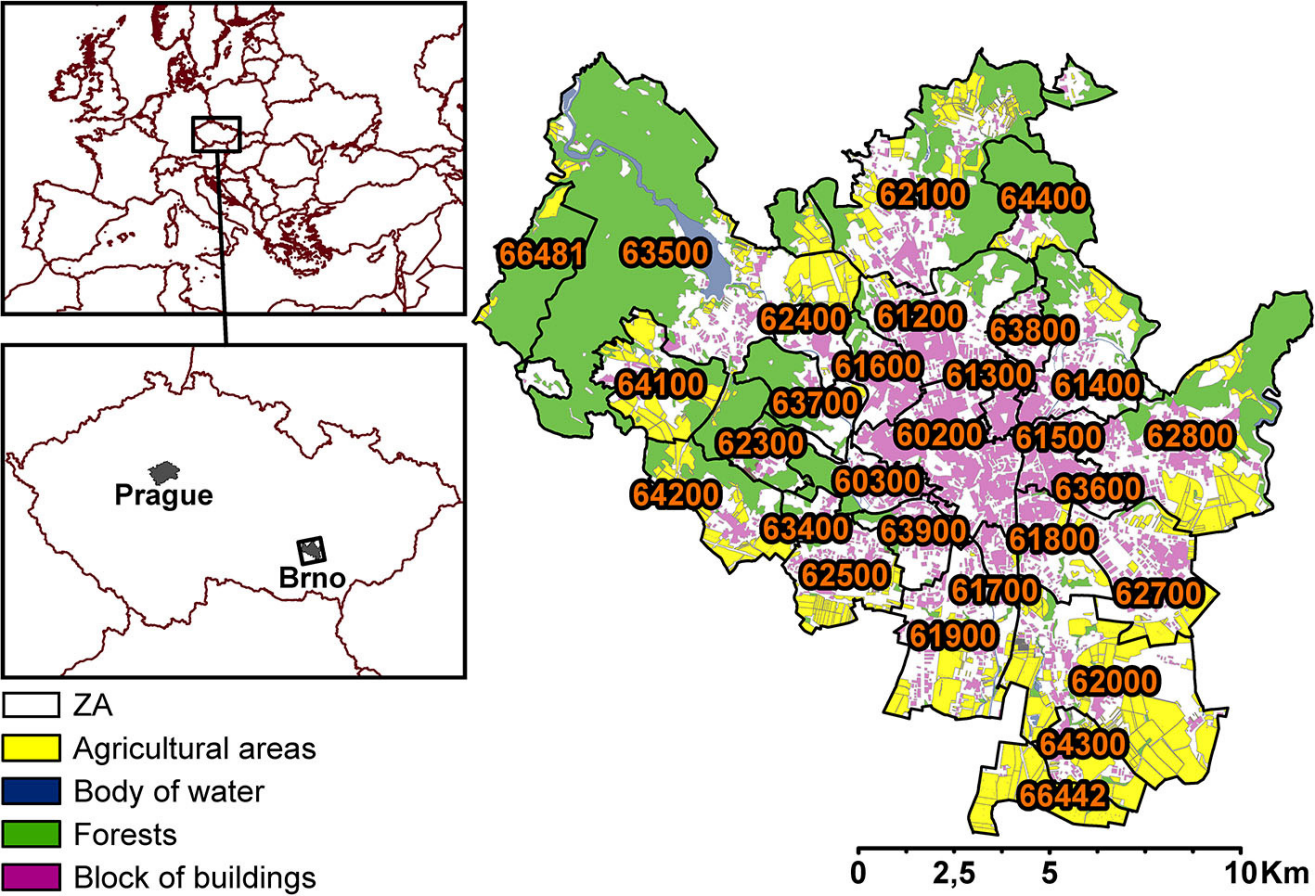
Division of ZAs (29) within the city of Brno (the labels are specific ZA codes), showing agricultural areas, bodies of water, forests, and blocks of buildings.

The city of Brno, situated in the South Moravian region, is an important node for land transportation between three capitals: Prague, Vienna, and Bratislava. With roughly 380,000 inhabitants and an area of 230 km^2^, the city of Brno is the second largest city in the Czech Republic (CZ). It is divided into 29 ZAs (median area = 5.1 km^2^; Figure 1). Prominent sources of air pollution today are public electricity and heat generation, land transportation, and local heating (25).

### Air quality data

The National AQ Monitoring Network in CZ is operated by the Czech Hydrometeorological Institute (CHMI), which measures various air pollutants, such as PM_10_, PM_2, 5_, NO_x_, heavy metals, and volatile organic compounds (26). The monitoring network consists of multiple AQ monitoring stations across the whole country. There are 12 mostly automated monitoring stations (described in Section 1 in Supplementary material) on the premises of the city of Brno. Data from these stations serve as the basis for modeled concentration maps (27). The methodology of preparation is described in CHMI annual reports (28). In brief, the measured data are collected for the whole country and serve as primary data for mapping. Secondary data sources providing comprehensive information for the region are in the form of dispersion models (mainly CAMx) based on reported emissions, traffic intensities, and meteorology. The modeling outcomes are reported as maps with a 1 × 1 km grid resolution. The used concentration maps of 5-year averages span from 2007 to 2011 and from 2014 to 2018 and contain concentrations of PM_2.5_, PM_10_, NO_2_, benzene, benzo(a)pyrene (B[a]P), Cd, Pb, Ni, and As. The used annual AQ data span from 2010 to 2018 and include concentrations of PM_2.5_, PM_10_, NO_2_, NO_X_, SO_2_, and benzene. Concentration maps comprising 1 × 1 km grids for the entire city of Brno were transformed into ZAs with weighted average concentrations of each pollutant using a geographic information system (ArcGIS Pro, ESRI, USA). The layer of ZAs was intersected by the 1 × 1 km grid layer with information about concentrations (more information in Section 2 in Supplementary material). The final concentration value was then calculated as a weighted average.

Of the 29 ZAs, two areas (ZA 66481 and 66442) were omitted since there are no inhabitants in these areas and they consist mostly of forests, fields, and meadows (see details in Section 3 in Supplementary material). The maps were prepared in the same manner for each relevant pollutant and each year. The AQ index was prepared by 0–1 standardization and by averaging all concentrations within each ZA. The concentrations of each pollutant were first averaged within each ZA. The average values were then normalized on a scale ranging from 0 (the least polluted) to 1 (the most polluted) for the purposes of data visualization in ZAs (see the Section 4 in Supplementary material).

### Health and population data

The selection of specific health conditions (HC) was based on the Health Risks of Air Pollution In Europe (HRAPIE) project (29). Health data for the city of Brno were obtained from the Institute of Health Information and Statistics of the Czech Republic (30). Health data were assigned for each region (ZA) based on permanent residency, reported by each patient. The data were fully anonymized for the relevant ZAs in the city of Brno. The studied time-period ranged from 2010 to 2018 to match the AQ data period. Data are expressed as the sum of annual occurrences of HC in each ZA. Each HC is characterized by a varying number of International Classification of Disease (ICD, 10^th^ version) codes. Data were standardized as the number of cases per 100,000 capita (in the respective age group). HCs, abbreviations of HCs, and a list of ICD-10 codes for each HC can be found in Table 1.

**Table 1:** HC with age groups, HC abbreviations, and respective codes under ICD-10.

**HC**	**Abbreviation**	**ICD-10**
Prevalence of acute and chronic bronchitis (0–19)	HC1	J20, J40, J41, J42, J44, J45.0, J45.9, J47, J68.0
Number of new cases of chronic bronchitis (20+)	HC2	J41, J42, J44.8
Number of new cases of asthma in asthmatic children (0–19)	HC3	J45.0, J45.9, J46
Hospitalization (respiratory diseases, all ages)	HC4	J00–J99
Hospitalization (respiratory diseases, 65+)	HC5	J00–J99
CVD hospitalization (including stroke, all ages)	HC6	I00–I99
CVD hospitalization (including stroke, 65+)	HC7	I00–I99

The Population and Housing Census 2011 (31) and the Populations of Municipalities of the CZ (32) collected by the Czech Statistical Office (CZSO) were used as sources of population data (33). Health data were also normalized in the same way as AQ data in Section 4 in Supplementary material.

### Additional indicators

Additional indicators of the urban exposome were also considered. They deal with built-up area indicators (BA) and socioeconomic indicators (SC). BA indicators focus on the characteristics of urban development (continuous urban fabric - CUF, discontinuous urban fabric - DUF, industrial and commercial area - ICA, road network - RN, green space – GS, noise levels - NL), SC indicators consist of education level (ED) and unemployment rates (UNP). A more detailed description of each indicator can be found in Section 5 in Supplementary material. The spatial resolution of all indicators was transformed to match the ZAs, but the temporal relation differed depending on each indicator. Absolute values of CUF, DUF, ICA, RN, and GS were adjusted for each ZA (area) and expressed as the area of indicator per square kilometer of ZA.

### Statistical analysis

The R programming language (34) was used for statistical analysis. Temporal trends in both AQ and health data sets were calculated by Kendall Tau correlation (35). Spearman's rank correlation coefficient was used to create a correlation matrix of all variables. Log-linear regression (Generalized Estimating Equation, GEE) was used to calculate associations between HCs and AQ while controlling for BA and SC (36, 37). GEE with Poisson distribution and logarithmic link function were used as follows:


$$\begin{array}{l} Log\left(HC_{i}\right)=\beta_{0}+\beta_{1}AQ_{k}+\beta_{2}A_{f}+\;\ldots \;+\beta_{x}A_{j} \end{array}$$

where *HC*_*i*_ denotes response variable (health condition) *i*, β denotes regression parameters, *AQ*_*k*_ denotes the predictor variable (air quality) *k*, and *A*_*f*_ denotes adjusting variable *f*. Relative risk (RR) for each predictor variable was calculated as follows:


$$\begin{array}{l} RR=\;{\left(e^{\beta_{1}}\right)}^{increment\;of\;change} \end{array}$$

where increment of change was based on the interquartile range (IQR, Section 6 in Supplementary material) of AQ predictors. GEEs were calculated as bivariate models for HCs and AQ indicators. These models were further adjusted by indicators of BA and SC in multivariate models. The false discovery rate method devised by Benjamini and Hochberg was used to adjust *p*-values and limit the rate of false positive results (38).

## Results

Descriptive statistics with time-series Kendal correlations between all studied pollutants and annual air pollution limits for the EU and recommendations from WHO are listed in Table 2 (3, 39). Mean values for all pollutants were within EU annual limits. Except for SO_2_, NO_X_, and arsenic, all pollutants showed a statistically significant decrease in concentration over the observed period. WHO health recommendations on annual air pollution limits were not met by PM_2.5_, PM_10_, NO_2_, and B[a]P.

**Table 2:** Descriptive statistics and Kendall correlation (KC) in the period 2010–2018, for air pollutants over the city of Brno and AQ limits.

**Pollutant**	**Unit**	**Mean ±SD**	**Median**	**KC**	**EU annual limit**	**WHO recommendation (annual)**
PM_2.5_	μg/m^3^	20.5 ± 2.53	20.1	−0.71^*^	25	5
PM_10_	μg/m^3^	26.2 ± 2.78	26.0	−0.61^*^	40	15
NO_2_	μg/m^3^	21.3 ± 6.00	20.8	−0.78^**^	40	10
NO_X_	μg/m^3^	37.6 ± 13.90	35.2	−0.17	–	–
SO_2_	μg/m^3^	4.6 ± 1.14	4.5	−0.22	20	40
Benzene	μg/m^3^	1.5 ± 0.35	1.5	−0.78^**^	5	1.7
B[a]P	ng/m^3^	0.9 ± 0.18	0.8	−0.86^**^	1	0.12
As	ng/m^3^	0.9 ± 0.10	0.9	0.07	6	6.6
Cd	ng/m^3^	0.3 ± 0.05	0.2	−1.00^****^	5	5
Ni	ng/m^3^	1.7 ± 0.54	1.6	−0.93^***^	20	25
Pb	μg/m^3^	8.1 ± 1.55	8.1	−1.00^****^	500	500

PM_2.5_; PM_10_; NO_2_; SO_2_; benzene – annual data (2010–2018), B[a]P; As; Cd; Ni; Pb – 5-year mean data (2007–2011 to 2014–2018).

* Significant p ≤ 0.05.

** Significant p ≤ 0.01.

*** Significant p ≤ 0.001.

**** Significant p ≤ 0.0001.

**Figure 2:**
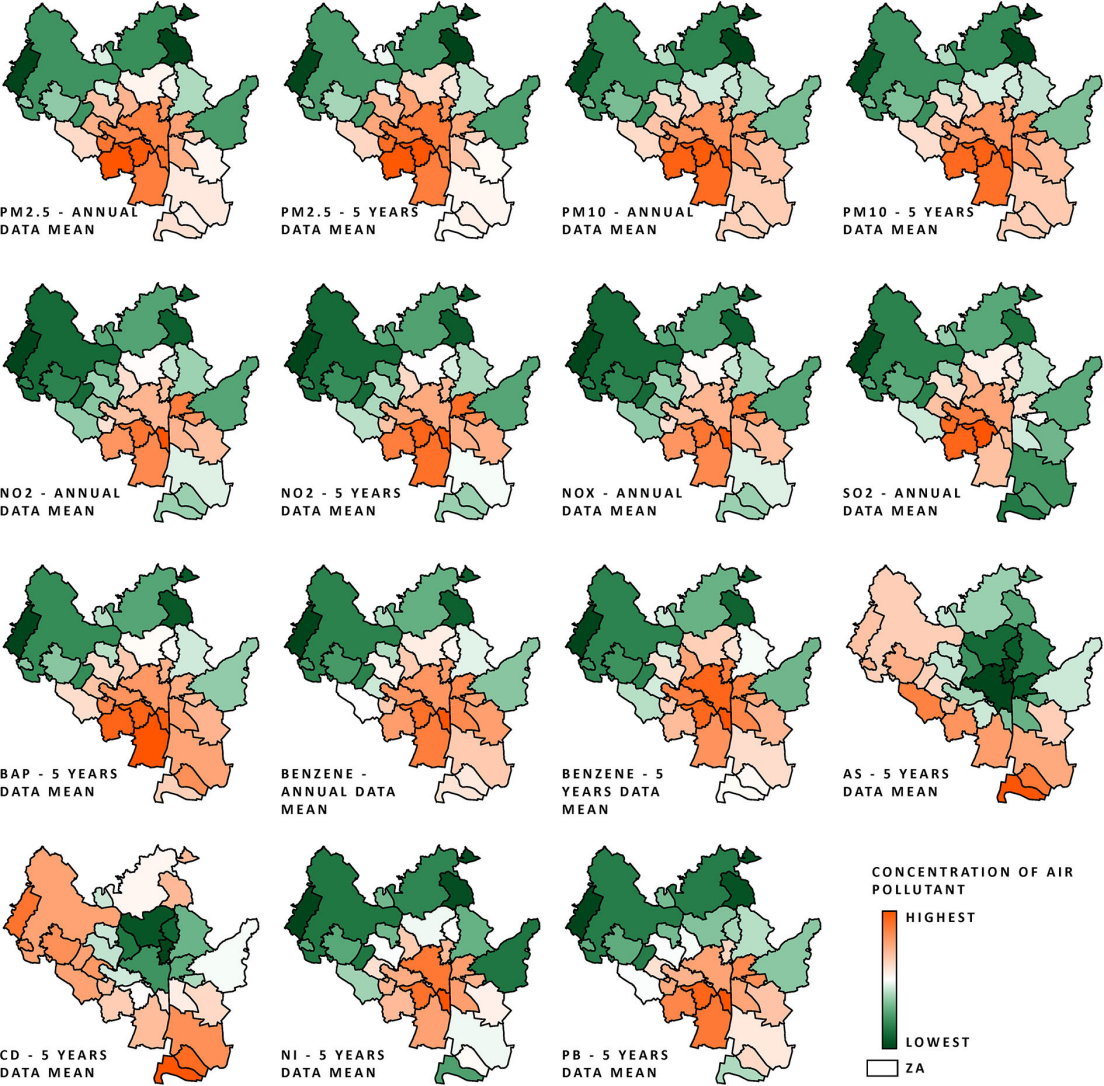
AQ data (normalized on a 0–1 scale; procedure described in Air quality and Section 4 in Supplementary material) of all pollutant concentrations within the city of Brno ZAs from the period 2010–2018 (annual data) and from the periods 2007–2011 to 2014–2018 (5-year mean data); the scale ranges from orange (highest air pollution) to green (lowest air pollution).

As shown in Figure 2, the areas with the lowest concentrations of the studied pollutants were mostly located in the northern parts of the city. Those areas mostly consist of forests, meadows, and residential areas, or are not inhabited at all. Locations with the highest modeled concentrations of the studied pollutants are situated around the city center and in the south. Both arsenic and cadmium exhibited considerably different distributions within the city limits in comparison to the other pollutants.

**Figure 3:**
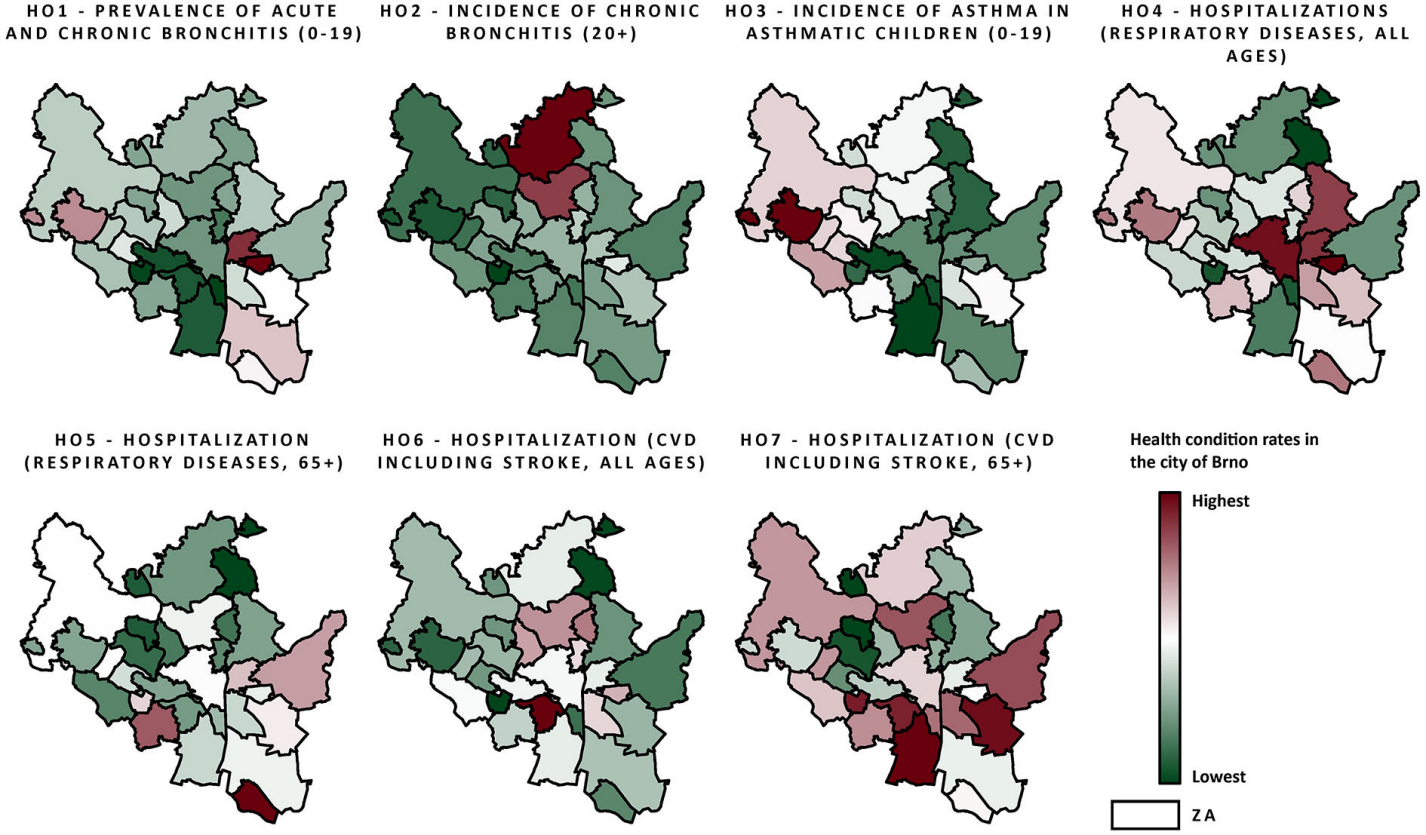
Health data (normalized on a 0–1 scale; procedure description in Air quality data and Section 4 in Supplementary material) within the city of Brno ZAs from the period 2010–2018, including all observed health conditions; the scale ranges from red color (highest rates) to green color (lowest rates).

Health conditions for the city of Brno in the years 2010–2018 are visualised in Figure 3. A summary is provided in Table 3, together with data for the whole of the Czech Republic and approximate data for EU/western cities (29, 40). Data on the prevalence and incidence of HCs do not differ much from national mean values. More pronounced differences can be seen in hospital admissions, where the city of Brno has lower admission rates in comparison to national rates. In comparison to EU/western cities, HC rates fluctuate depending on the specific diagnosis. Bronchitis in adults (HC2) was roughly two-fold higher in the city of Brno. Hospital admission rates were, however, generally similar to those in the EU, although EU data were approximated according to hospital discharges and were not age-specific.

**Table 3:** Descriptive statistics and Kendall correlation (KC) for the time series 2010–2018, with significance levels for HCs for the city of Brno, CZ, and EU/western countries (29, 40).

**HC**	**City of Brno (per 100,000 capita)**			**CZ (per 100,000 capita)**			**EU health data** **(per 100,000 capita)**
	**Mean ±SD**	**Median**	**KC**	**Mean ±SD**	**Median**	**KC**	
HC1	12,294 ± 3,119	11,832	−0.56^*^	11,233 ± 540	11,227	−0.50^*^	18,600
HC2	699 ± 437	614	−1.00^****^	694 ± 190	642	−1.00^****^	390
HC3	1,282 ± 424	1,257	−0.64^*^	1,287 ± 248	1,280	−0.93^***^	3,500–4,900
HC4	1,219 ± 202	1,210	0.00	1,328 ± 49	1,303	0.00	1,407^a^
HC5	2,377 ± 860	2,347	0.83^***^	2,899 ± 466	2,956	0.89^***^	
HC6	2,211 ± 449	2,172	−0.78^**^	2,706 ± 106	2,703	−0.83^***^	2,416^a^
HC7	8,982 ± 1,596	8,946	−0.39	11,232 ± 230	11,198	−0.11	

* Significant ^p^ ≤ 0.05.

** Significant ^p^ ≤ 0.01.

*** Significant ^p^ ≤ 0.001.

**** Significant ^p^ ≤ 0.0001.

a Approximated as hospital discharges; KC - Kendall correlation; HC1 - Prevalence of acute and chronic bronchitis (0–19); HC2 - Incidence of chronic bronchitis (20+); HC3 - Incidence of asthma in asthmatic children (0–19); HC4 - Hospitalization (respiratory diseases, all ages); HC5 - Hospitalization (respiratory diseases, 65+); HC6 - CVD hospitalization (including stroke, all ages), HC7 - CVD hospitalization (including stroke, 65+).

Respiratory hospitalizations in the 65+ age-group (HC5) increased sharply in the observed period with a high level of significance. The trend in respiratory (HC4) and CVD-related hospitalizations (HC7) in all ages declined or stagnated, respectively, although neither result was statistically significant. The remaining HC trends decreased, and these decreases were statistically significant. On the national level, correlations between trends were comparable to those for the city of Brno, although the actual strengths of the correlations differed.

Health conditions were weakly to moderately correlated (*r* < 0.5) among each other, with the strongest correlation (*r* = 0.44, *p* ≤ 0.0001) between the incidence of bronchitis (HC2) and the incidence of asthma (HC3). A similar strength of correlation was also found between respiratory hospitalizations (HC4, HC5, *r* = 0.42, *p* ≤ 0.0001), while a weaker correlation was found between CVD-related hospitalizations (HC6, HC7, *r* = 0.32, *p* ≤ 0.0001). The correlation matrix of all HCs can be found in Section 7 in Supplementary material.

AQ indicators were generally moderately (r ≥ 0.5) to strongly (r ≥ 0.8) correlated with each other; see Section 7 in Supplementary material. NO_x_ showed a strong correlation (r ≥ 0.9) with NO_2_ and thus was excluded from further analysis. Likewise, B[a]P, Ni, and Pb were eliminated due to strong intercorrelation (*r* ≥ 0.9) with PM_10_. The remaining heavy metals (As, Cd) were also excluded due to their ambient air concentrations, which were well below safety limits and their declining or stagnating trends.

**Figure 4:**
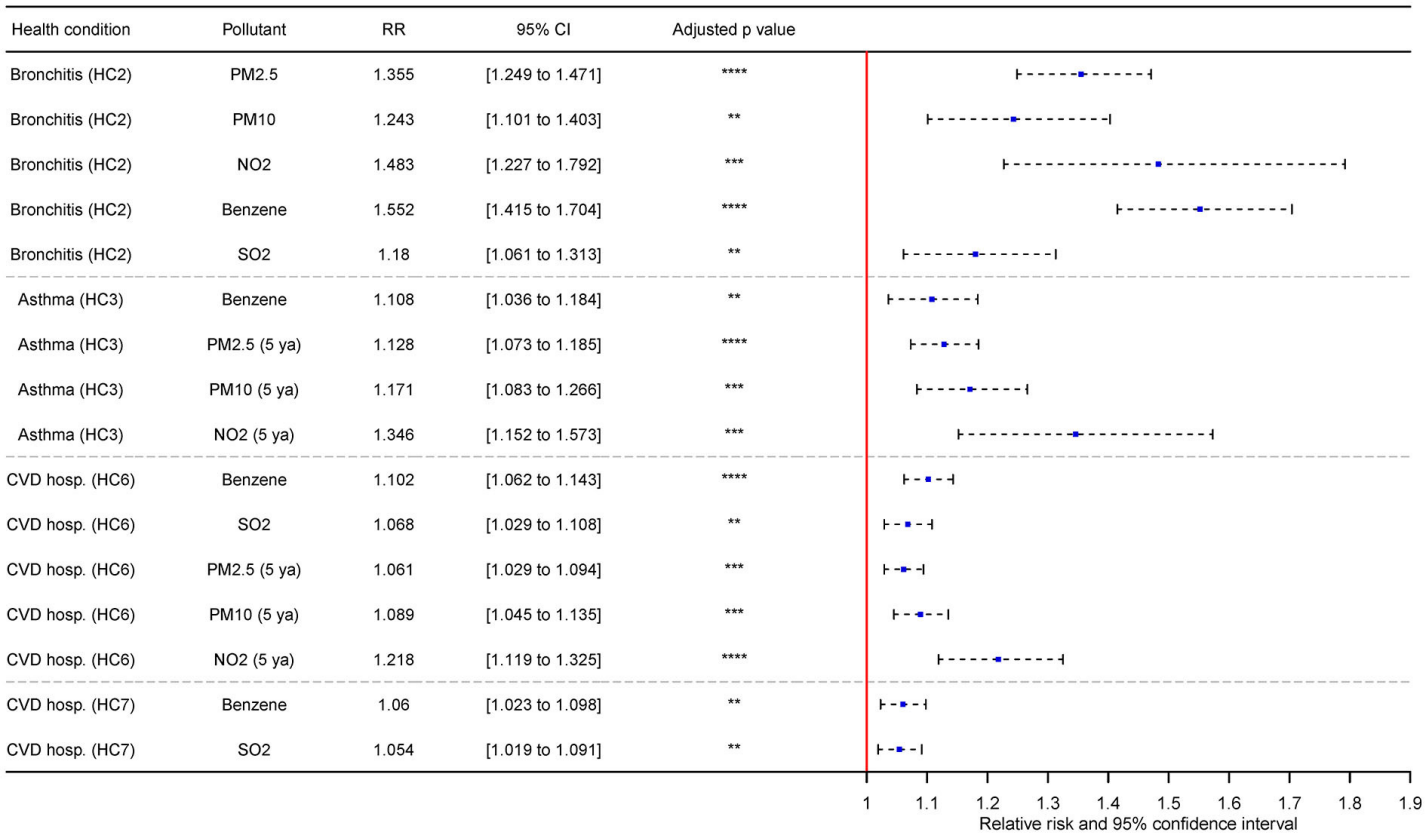
Selected associations between multivariate regression of HCs and AQ adjusted by BA and SC indicators with adjusted *p*-values and adjustment for changes based on IQR (**significant *p* ≤ 0.01; ***significant *p* ≤ 0.001; ****significant *p* ≤ 0.0001; HC2 - Incidence of chronic bronchitis (20+); HC3 - Incidence of asthma in asthmatic children (0–19); HC6 - CVD hospitalization (including stroke, all ages), HC7 - CVD hospitalization (including stroke, 65+); 5 ya – 5-year mean).

Selected results from adjusted models are shown in Figure 4; all significant associations of bivariate and adjusted models are listed in Section 8 in Supplementary material. The incidence of bronchitis (HC2) showed the strongest association with benzene, exhibiting an RR value of 1.759 CI (1.597–1.937). This association was overall the most salient in comparison to all other associations. Weaker but still considerable associations were also found with PM_10_, PM_2.5_, or NO_2_. After the adjustment, HC2 was again strongly associated with benzene [RR = 1.552 CI (1.415–1.704)] and similarly as in bivariate models, PM_2.5_, PM_10_, and NO_2_ were also strongly associated with HC2. Weaker, but still significant association was also found with SO_2_.

Incidence of asthma in children (HC3) was strongly associated with benzene (5-year average) RR = 1.614 CI (1.406–1.853). Weaker, yet still strong association was also observed in PM_10_. After the adjustment, HC3 was the most notably associated with NO_2_ [5-year average; RR = 1.346 CI (1.152–1.573)]. The strength of effect of the remaining significant pollutants (benzene, PM_10_) was diminished in comparison to the results of bivariate models. PM_2.5_ was also newly introduced as significant on the level comparable to PM_10_. In bivariate models, SO_2_, NO_2_ and benzene (5-year average) exhibited reversed association with HC3, but in adjusted models, these associations we no longer significant, or the effect of pollutants was shifted. The associations between bronchitis (HC2)/asthma (HC3) and AQ were the most prominent in comparison to the respiratory and CVD-related hospitalizations.

Respiratory hospitalizations in all ages (HC4) were associated with NO_2_ (5-year average), albeit relatively weakly [RR = 1.044 CI (1.003–1.088)]. The similar strength of association was also observed with benzene and HC4. No air pollutant was further significant with HC4 after the adjustment for BA and SC. Contrary to expected results, respiratory hospitalizations in elderly (HC5) was positively associated with AQ indicators. This was also true in adjusted models.

The remaining CVD-related hospitalizations (HC6, HC7) were associated in bivariate models with PM_2.5_, PM_10_, NO_2_, benzene, and SO_2_. The strongest association for HC6 was with NO_2_ (5-year average) – specifically, RR = 1.162 CI (1.113–1.213) and RR = 1.12 CI (1.079–1.163) for HC7. The difference between significant models in the case of HC6 and HC7 was relatively small, with the lowest significant effect of PM_2.5_ on HC7 – specifically, RR = 1.032 (1.002–1.063). After the adjustment for BA and SC, the effect of most associations in was slightly diminished in comparison to bivariate models, and PM_2.5_, PM_10_ and NO_2_ were no longer significant for HC7.The strongest association was found between HC6 and NO_2_ [5-year average; RR = 1.218 CI (1.119–1.325)] which is slight increase in comparison to bivariate models.

Only SO_2_ was significant for the prevalence of bronchitis (HC1) in bivariate models. However, SO_2_ in this association exhibit reversed relation with the HC1, and after the adjustment, no significant model was found for HC1. Most of the associations among all HCs were characterized by highly significant *p*-values and adjusted *p*-values (*p* ≤ 0.0001). These were often found in CVD hospitalization HCs (HC6, HC7) or the incidence of bronchitis (HC2).

## Discussion

We investigated respiratory and CVD health conditions from 2010 until 2018. Bronchitis, one of the investigated HCs, was found to be following a declining trend in the city of Brno as well as on the national scale. This trend was observed globally over the past decade (41). Throughout the 2000s, there was a slowly declining trend in respiratory-related hospital discharges in all age groups (approximated as hospital admissions for respiratory diseases) in the Czech Republic (40). In contrast, there was no trend over the observed period (2010–2018) on the national or the city of Brno level. However, an increasing trend was found for the same HC but in the age group 65+. The aging of the population might have contributed to this increase (42). The same driver was inevitably affecting CVD-related hospitalizations. However, there was an evident declining trend in CVD-related hospitalizations (across all age groups), which is in line with European trends since the mid-2000s (43). A weaker and non-significant trend was also observed in the same HC and the 65+ age group. In comparison to respiratory and cancer-related mortalities, CVD is often proportionally more prominent in Central and Eastern Europe, which is also true for the Czech Republic (44). At the same time, the Czech Republic is one of the countries with the lowest rates of unattended medical examinations (44), one which has improved on the life expectancy of its citizens in past years (45), and in which there is relatively little social inequality in the provision of healthcare (46). These indicators point toward improvements in healthcare in the Czech Republic, which may have contributed to the decline in CVD-related hospitalizations.

The impact of AQ on human health is often investigated with a primary focus on PMs and NO_2_ since these pollutants are abundant in the urban environment. We investigated long-term annual, and 5-year mean levels of these and additional pollutants in the city of Brno. In comparison with other Czech cities, the city of Brno is among the more polluted conurbations (Section 9 in Supplementary material). On the brighter side, in most cases, there was a significant declining trend in ambient atmospheric pollutant levels over the observed period. AQ is a known contributor to increasing respiratory and CVD health problems (39), and there were notable improvements in most of the observed AQ indicators. This may also be a reason for the decrease in the rates of some HCs over the observed period.

In this study, we identified PMs, NO_2_, benzene, and SO_2_ as potential risk factors in respiratory and CVD-related HCs. The most prominent association was found between benzene and the incidence of bronchitis (HC2). Other strong associations were between NO_2_ and the incidence of bronchitis (HC2), or between NO_2_ and CVD-related hospitalizations (HC6). The remaining pollutants had a weaker but still significant negative impact on the incidence of bronchitis (HC2), the incidence of asthma (HC3), and CVD-related hospitalizations (HC6, HC7).

Adverse associations of benzene with respiratory problems were observed in the past. Oftedal et al. (47) reported an RR of 1.10 CI (1.04–1.17) for benzene associated with respiratory hospitalizations in a time-series study in Norway. Penard-Morand et al. (48) found an association between exposure to benzene and a children's asthma odds ratio (OR) of 1.25 CI (1.08–1.43) in a study covering six French cities, and Hirsch et al. (49) reported an OR of 1.16 (1.04–1.29) linking benzene and bronchitis in a German cross-sectional study. Further evidence associating benzene with adverse effects on the respiratory system was also reported in recent studies by Idavain et al. (50) and Ran et al. (51), and in the meta-analysis by D'Andrea and Reddy (52).

The effect of NO_2_ on CVD was estimated in the meta-analysis (based on seven studies) by Pranata et al. (53) as a hazard ratio (HR) of 1.15 CI (1.02–1.29). Similar results were reported by Huang et al. (54), who, on the basis of 20 studies, estimated the HR for CVD mortalities as 1.11 CI (1.07–1.16). Other current studies reported an OR of 1.17 CI (1.01–1.35) on lag day 2 (55) or an RR of 1.05 CI (1.02–1.08) on lag day 1 (56), although these studies worked with short-term exposure.

Associations between SO_2_ and respiratory or CVD HCs were documented in previous epidemiological studies. Respiratory hospital admissions in children linked to SO_2_ [RR = 1.28 CI (1.22–1.33)] were reported in the Brazilian metropolitan area (57). Vascular damage in young adults was associated with long-term exposure to SO_2_, representing an excess risk of 5.26% CI (0.09–10.43) (58) and Dong et al. (59) reported an OR of 1.11 CI (1.04–1.18) linking SO_2_ and hypertension in the Chinese population.

Many studies have reported a link between PMs and respiratory and CVD diseases in past years (8, 60–63). Our results are mostly consistent with the literature. However, it is important to note that in the case of benzene, for example, current literature mostly deals with respiratory HCs in children and that epidemiological evidence for adults is limited. A similar issue arises in CVD-related hospitalizations, where characteristics of HCs are not the same across studies, although hospitalizations might be well defined by ICD. Perhaps the most significant deviation from literature lies in CVD-related hospitalizations. Vaduganathan et al. (64) reported an increased risk of CVD-related hospitalizations linked with PM_10_ [RR = 1.004 CI (1.002–1.006)]. Similarly, Larrieu et al. (65) reported an excess risk = 0.7% CI (0.1–1.2) of all-case CVD-related hospitalizations (all ages) and excess risk = 1.1% CI (0.5–1.7) of all-case CVD-related hospitalizations (65+) after exposure to PM_10_. Complementary results were also published by other authors (12, 66, 67). Previously reported results are lower in comparison to our results, as we report risks with respect to CVD-related hospitalizations equal to RR = 1.089 (1.045–1.135) for PM_10_ and RR = 1.061 (1.029–1.094) for PM_2.5_.

We observed different strength of associations of AQ indicators in bivariate and multivariate models. AQ indicators are to a various degree correlated with multiple other indicators of AQ, BA, SC (Section 7 in Supplementary material), and possibly with other unmeasured pollutants. Thus, after the adjustment for BA and SC, we can observe, that the effect of AQ can be altered in comparison to the bivariate models (e.g., benzene and HC2). Meaning that some variability in the HC3 can be attributed to the BA and SC.

Generally, it seems that PM_2.5_ is slightly more associated with certain HCs in comparison to PM_10_. This was demonstrated for example by Lu et al. (68). However, in our study, PM_10_ was slightly more associated with HC3 and HC6 in comparison to PM_2.5_. To a degree, similar results were also published by Darrow et al. (69), de Keijzer et al. (70), Janssen et al. (71), and Sicard et al. (72). This can be partially explained by differences in emission sources between different geographical regions. This then makes direct intercomparison of PMs associations with HCs challenging. Another likely explanation may stem from the fact that the RRs of both pollutants are calculated from IQR, which differs between those two in the studied period.

There also appeared other deviations in the associations between AQ and respiratory hospitalizations (HC5), the incidence of asthma (HC3), and the prevalence of bronchitis (HC1). These associations attribute the positive effects on the mentioned HCs to AQ. In the case of HC3 and after adjustment for BA and SC, AQ appeared as a risk factor, while in the case of HC1 the associations were no longer significant. However, in case of HC5, the reversed effects persist even after the adjustment. Similar inconsistency in the results was observed in previous studies. For example, de Keijzer et al. (70) reported that NO_2_ was inversely related to mortality in unadjusted but also in adjusted models as well as conflicting results for greenness indicators. Carey et al. (73) reported NO_x_ and PM_2.5_ as being positively related with stroke and myocardial infarction, and Luginaah et al. (74) found the same unconventional relationship between CO and respiratory hospitalizations in males 65+ on lag days 1 and 2. Both studies reported significant trends, even after adjustment for BMI, sex, smoking, and age (first study) and meteorological parameters (second study). Mixed results on respiratory HC were also reported by Pun et al. (62) and Pope et al. (75). In the city of Brno, the elderly people tend to live in the northern parts of the city (Section 10 in Supplementary material), while worse AQ is situated more around south and central parts. This may have negative impact the results for HC5. And while previous studies dealing with similar topic might have associated AQ with negative impacts on respiratory hospitalizations, our results are mostly inconclusive or mixed for this HC.

### Limitations

One of the limitations of our study may be the time resolution of annual and 5-year mean concentrations; some other studies were based on the daily mean, as some of the HCs in question may be more affected by short-term spikes in AQ. Spatial resolution probably plays some role in deriving the relationships between AQ and health. The health data we used in this study represents the most accurate information on health situation in city scale. However, this dataset is limited in the spatial resolution, which is restricted to the ZAs, but finer resolution could be beneficial for example for HC3. The incidence of bronchitis (HC3) is high in western parts of the city, which have relatively low air pollution and high proportions of GS compared to other districts. However, the ZAs might be too coarse to return a specific picture of actual associations. This is probably related to the fact that the inner city has a more uniform nature within ZAs in comparison to city outskirts, where the landscape within ZAs is significantly more varied. Although outskirt ZAs have more GS, inhabitants are not equally dispersed in the ZA, but rather concentrated in secluded residential areas. Though ZA resolution may potentially be sufficient on the national level (76), it may not be detailed enough on the city scale level and thus leave room for potential improvement in the future.

It is also important to acknowledge the fact that associations do not reflect causal associations, but more of a general tendency within the observed population. As such, the used indicator may be correlated with several unobserved variables. This is especially true for studies with an ecological design. Population data and the ecological concept of a given study will always be limited by the inability to include other accompanying variables possibly affecting the studied diseases. For example, bronchitis may also be caused by exposure to various chemical irritants in tobacco smoke, and other agents (77). The spatial resolution of AQ indicators (1 × 1 km grid) is based on dispersion models (28), which also brings uncertainties. These factors should be considered as limitations pertaining to this study.

### Future research

We propose continuous assessment of the impacts of AQ on human health in this city-scale approach. We already managed to implement this approach in the city of Brno, where this assessment will be carried out over the next years in collaboration with the city hall. We will also include another predictors of urban exposome such as light pollution (78), meteorological conditions (79) or other socio-economic parameters. And while so far, we are limited in spatial resolution of ZAs, this limitation may be surpassed in the future, increasing the accuracy of the results. When appropriated, the outputs of this study could be of use to local policy makers with respect to the management of health risks. Continuous assessment of health risks over the following years could uncover potential risks associated with exposure to poor AQ, but it also may point out advantages of improving AQ (80–82). This may also be advantages for concerned citizens as this will also be publicly available bringing science to citizens.

## Conclusion

Revealing the impact of AQ on human health is not an easy task, especially when many factors need to be considered. In this study, we approached this problem at the city scale level with the intention of using spatial and temporal data resolutions that were as high as possible. We managed to increase the spatial resolution of all data sets to the level of ZAs. We found that several air pollutants (benzene, PMs, NO_2_, and SO_2_) were associated with respiratory and cardiovascular HCs in the city of Brno. We demonstrated the need for continual improvements in AQ and the usefulness of detailed data collection.

## Data availability statement

The datasets are not publicly available as they are subject to licenses and restrictions by the Institute of Health Information and Statistics of the Czech Republic. Access requests should be directed to http://www.uzis.cz/.

## Author contributions

LŠ: formal analysis, investigation, writing—original draft, and visualization. PG: statistical analysis, critically revised the study, and visualization. JK: statistical analysis, and critically revised the study. OM and TJ: writing—review and editing. PČ: conceptualization, coordination, resources, writing—review and editing, supervision. All authors read and approved the final manuscript.

## Funding

Authors thank Research Infrastructure RECETOX RI (No. LM2018121) and project CETOCOEN EXCELLENCE (No. CZ.02.1.01/0.0/0.0/17_043/0009632) financed by the Ministry of Education, Youth and Sports for supportive background. This work has received funding from the European Union's Horizon 2020 research and innovation program under grant agreements Nos. 857340, 874627, and 689443. This work was supported by the European Union's Horizon 2020 research and innovation program under grant agreement No. 857560. This publication reflects only the author's view, and the European Commission is not responsible for any use that may be made of the information it contains.

## Conflict of interest

The authors declare that the research was conducted in the absence of any commercial or financial relationships that could be construed as a potential conflict of interest.

## Publisher's note

All claims expressed in this article are solely those of the authors and do not necessarily represent those of their affiliated organizations, or those of the publisher, the editors and the reviewers. Any product that may be evaluated in this article, or claim that may be made by its manufacturer, is not guaranteed or endorsed by the publisher.
