# Supplementary material for: City-scale assessment of long-term air quality impacts on the respiratory and cardiovascular health
Source: Front Public Health. 2022 Nov 10;10:1006536. doi: 10.3389/fpubh.2022.1006536 (PMC9687097; doi:10.3389/fpubh.2022.1006536)
Supplement: Supplementary file 1 [file Data_Sheet_1.docx]

*Supplementary Material*

# AQ monitoring stations in the city of Brno

AQ monitoring stations on the premises of the city of Brno are shown in the **SI Figure 1**. More information on each station is in **SI Table 1**.


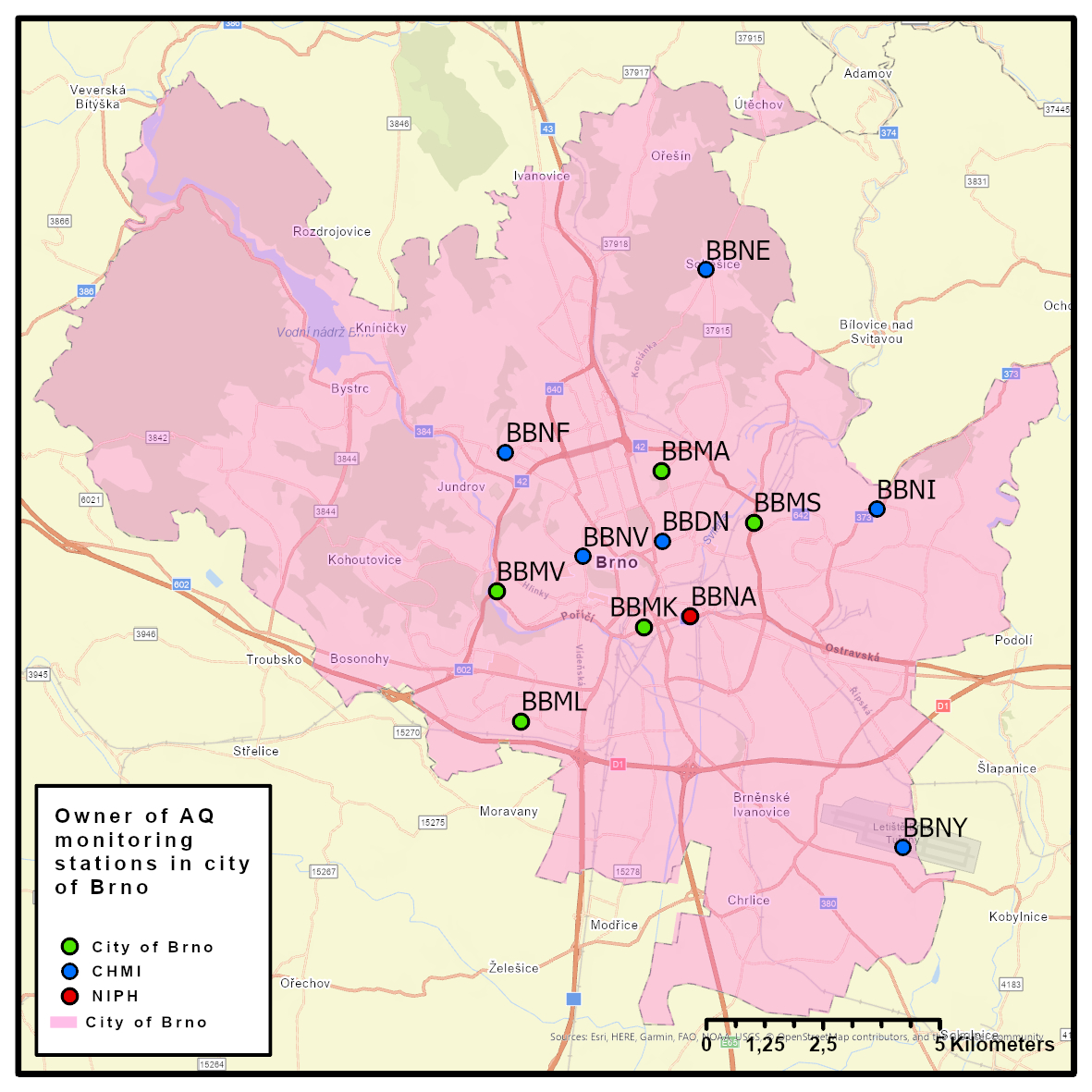


SI Figure 1 Map of all AQ monitoring stations on premises of the city of Brno (CHMI, 2021), (CHMI – Czech Hydrometeorological Institute, NIPH - National Institute of Public Health).

SI Table 1 Overview of AQ monitoring stations on premises of the city of Brno with type, zone characteristics, owner, position, and altitude.

| **Station** | **Type** | **Zone** | **Characteristics** | **Code** | **Owner** | **LAT.** | **LONG.** | **Altitude (m)** |
| --- | --- | --- | --- | --- | --- | --- | --- | --- |
| Brno Turany | Background | Suburban | Residential | BBNY | CHMI | 49.1489717 | 16.6962167 | 241 |
| Brno Uvoz | Traffic | Urban | Residential | BBNV | CHMI | 49.1980897 | 16.5936431 | 235 |
| Brno Lisen | Background | Urban | Residential | BBNI | CHMI | 49.2132111 | 16.6780242 | 340 |
| Brno Vystaviste | Traffic | Urban | Residential | BBMV | Brno | 49.1896208 | 16.5695381 | 202 |
| Brno Masna | Background | Urban | Commercial | BBNA | NIPH | 49.1888314 | 16.6270008 | 214 |
|  |  |  | Residential |  |  |  |  |  |
| Brno Svatoplukova | Traffic | Urban | Residential | BBMS | Brno | 49.2081603 | 16.6425172 | 213 |
| Brno Lany | Background | Suburban | Residential | BBML | Brno | 49.1652603 | 16.5808125 | 228 |
|  |  |  | Natural |  |  |  |  |  |
| Brno Zvonarka | Industrial | Urban | Commercial | BBMK | Brno | 49.1858825 | 16.6136608 | 200 |
| Brno Arboretum | Background | Urban | Residential | BBMA | Brno | 49.2160872 | 16.6138364 | 250 |
|  |  |  | Natural |  |  |  |  |  |
| Brno Detska nemocnice | Background | Urban | Residential | BBDN | CHMI | 49.2027244 | 16.6162872 | 225 |
|  |  |  | Commercial |  |  |  |  |  |
| Brno Sobesice | Background | Suburban | Residential | BBNE | CHMI | 49.255543 | 16.620497 | 380 |
| Brno Kroftova | Traffic | Urban | Residential | BBNF | CHMI | 49.216472 | 16.567761 | 235 |

# Calculation of weighted average of AQ data in ZAs

Following procedure (**SI Figure 2**) was carried out, to calculate weighted averages of AQ data in the city of Brno ZAs:

1. Required data: ZAs polygons, AQ data polygons 1×1 km for the city of Brno
2. Calculate the surface area of each ZA in the ZAs layer
3. Intersect (operation) ZA layer with AQ layer (1×1 km grid) for the city of Brno
4. Calculate the surface area of newly generated polygons created after intersect operation
5. Divide (field calculator) area of newly created polygons within ZA by total area of ZA (“partial area”)
6. Multiply (field calculator) partial area by the concentration of selected pollutant (“index”)
7. Dissolve (operation) all polygons into original ZA polygons with statistics option “index” and sum and statistic
8. Newly created layer contains weighted averages of selected air pollutant in each ZA


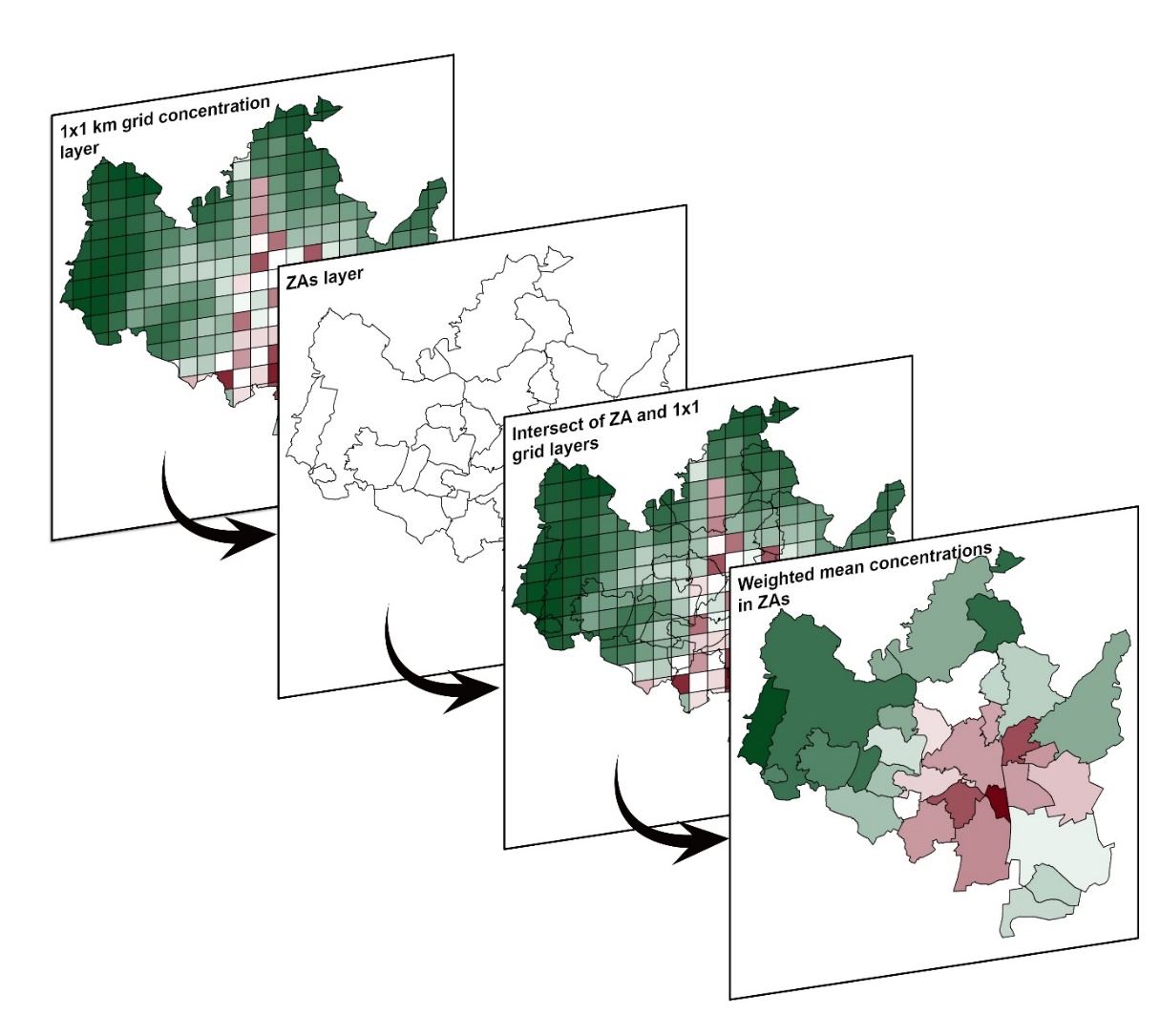


SI Figure 2 Schematic depiction of intersecting and dissolve operations in ArcGIS PRO

# Excluded ZAs in the city of Brno

**SI Figure 3** shows a map of two excluded ZAs (66481, 66442) which holds no households and consists mostly of forests or agricultural areas.


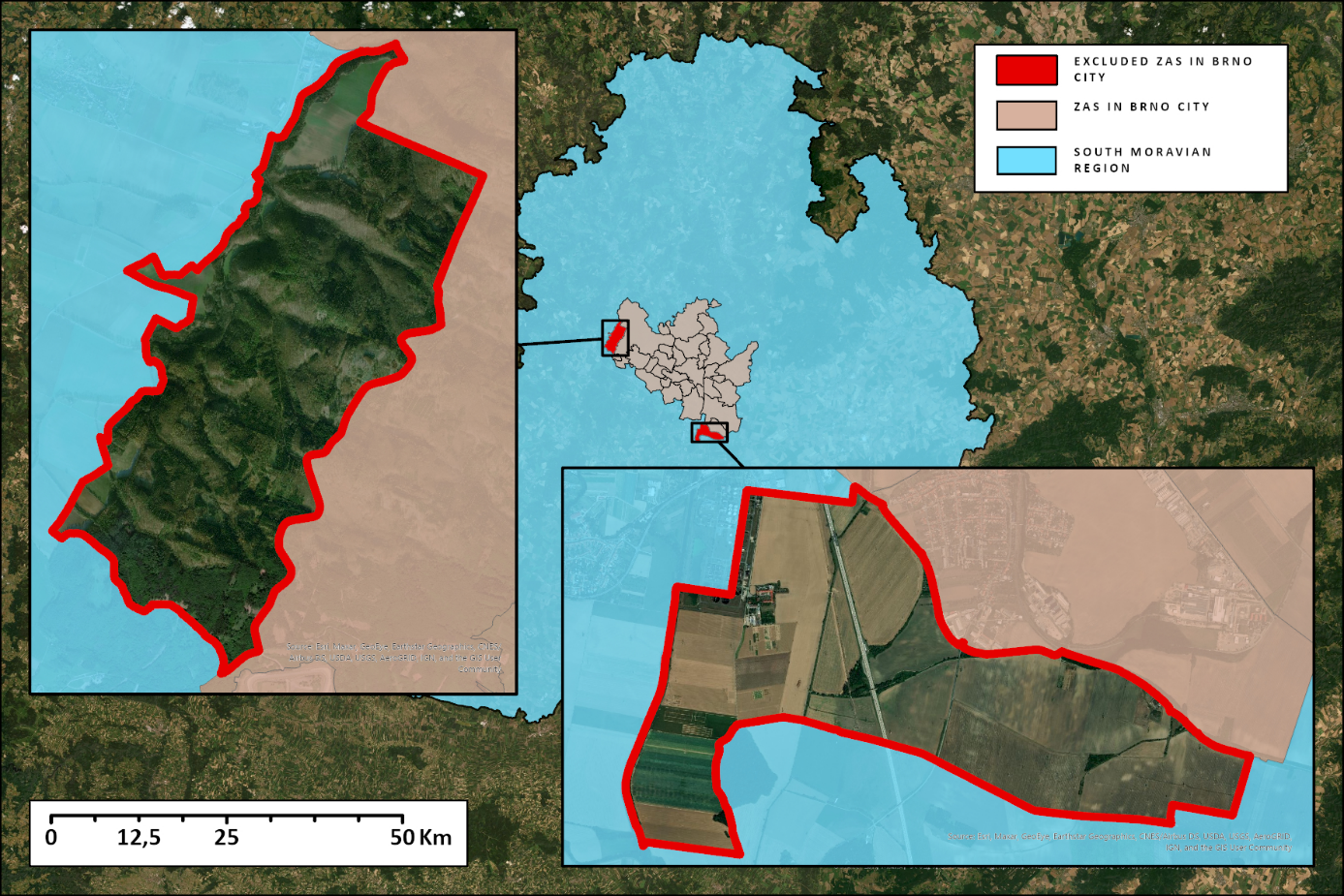


SI Figure 3 Excluded ZAs in the city of Brno

# Data normalization (0-1 scale)

AQ and health data were normalized on a 0-1 scale for direct visual comparison within each data group. Normalization was done according to the following steps:

1. Weighted mean values for AQ/health data were at first aggregated into ZAs means for years 2010 – 2018.
2. Aggregated AQ/health data were then transformed by the following equation:

$$x´=(x-x_{min})/(x_{max}-x_{min})$$

# Additional variables

An overview of all adjusting variables is in **SI Table 2.** All listed variables were used as ArcGIS PRO (.shp) data layers and suitably transformed/recalculated for each the of ZAs in the city of Brno. Data were downloaded/obtained from Copernicus Earth Observation Programme, CORINE Land Cover (CLC, 2018), Czech Office for Surveying, Mapping and Cadastre (COSMC, 2021), Global Human Settlement Layer (Florczyk *et al.*, 2019), GeoFabrik open street map data extracts (GeoFabrik, 2016), online data portal of the Hall of the city of Brno (*Data Brno*, 2022), and Population and Housing Census 2011 (CZSO, 2020).

SI Table 2 Additional variables with abbreviations and reference

| **Indicator** | **Abbreviation** | **Data source** |
| --- | --- | --- |
| Discontinuous urban fabric | DUF | (CLC, 2018), (Kosztra *et al.*, 2017) |
| Continuous urban fabric | CUF | (CLC, 2018), (Kosztra *et al.*, 2017) |
| Industrial and commercial area | ICA | (CLC, 2018), (Kosztra *et al.*, 2017) |
| Road network | RN | (GeoFabrik, 2016) |
| Build up density | BD | (Florczyk *et al.*, 2019) |
| Urban green space | GS | (COSMC, 2021) |
| Noise levels | NL | (*Data Brno*, 2022) |
| Unemployment rate | UNP | (CZSO, 2020) |
| Education | ED | (CZSO, 2020) |

## Build up area indicators

### Continuous urban fabric (CUF)

At least 80% of the total area is covered by impermeable artificial structures and road network. This definition includes urban city centres, public services, local governments, commercial or industrial areas with the surface under 25 ha, parking lots, green space with less than 20% coverage (Kosztra *et al.*, 2017). Data are related to the situation in 2018 and originally apply to specific polygons on the premises in the city of Brno. Polygons were intersected with the ZAs layer and dissolved into each ZAs. The indicator was then expressed as the area (km^2^) of CUF in each ZA per km^2^.

### Discontinuous urban fabric (DUF)

From 30% to 80% of the total area is covered by impermeable artificial structures and road network. This area contains proportionally more green space (gardens, public green space…) in comparison to CUF. This definition includes housing estates, residential areas, suburbs, small villages, blocks of flats, transport network, parking lots, sports areas under 25 ha, public places, educational and health buildings, market places under 25 ha, gardens, public green space (Kosztra *et al.*, 2017). Data are related to the situation in 2018 and originally apply to specific polygons on the premises in the city of Brno. Polygons were intersected with the ZAs layer and dissolved into each ZAs. The indicator was then expressed as the area (km^2^) of DUF in each ZA per km^2^.

### Industrial and commercial area (ICA)

Most of the area is covered by artificial surfaces (asphalt, concrete, tarmacadam…) and buildings with a minimum of vegetation. This definition includes transportation network, research and development areas, fire stations, fair sites, livestock farms, military areas, power plants, wastewater treatment plants, transformers, shopping malls, exposition centres, hospitals, schools, universities, parking lots, industrial brownfields, telecommunication networks etc. (Kosztra *et al.*, 2017). Data are related to the situation in 2018 and originally apply to specific polygons on the premises in the city of Brno. Polygons were intersected with the ZAs layer and dissolved into each ZAs. The indicator was then expressed as the area (km^2^) of ICA in each ZA per km^2^.

### Road network (RN)

The road network data layer (GeoFabrik, 2016) was derived from OpenStreetMap (OSM, 2022) of roads, highways, sidewalks, underpasses, footpaths, horse trails, forest roads etc. The layer for Europe in 2016 was reduced only to the highways and roads on the premises of the city of Brno.

### Green space (GS)

Data are originating from the digital geographic model for the Czech Republic derived from the cartographic map on a scale of 1:50,000. The original data set contains a thematic area of municipalities, borders of territorial units, cultural and agricultural structures, road network, electrical grid, vegetation and surface, blue area, and terrain relief. Thematic area of vegetation includes forests, meadows, pastures, peat bogs, swamps, marches, gardens, parks, vineyards, orchards and hop gardens (COSMC, 2021). One layer was created by the combination of forests, meadows, gardens, and parks data sets for the area of the city of Brno and subsequent intersection with the ZAs layer. Finally, a green area was dissolved into each ZAs. The indicator was then expressed as the area (km^2^) of GS in each ZA per km^2^.

### Noise levels

The data on noise levels were collected by Ministry of Health of the Czech Republic in the year 2017. Polygon layer for the city of Brno was downloaded as publicly available information from online data portal of the Hall of the city of Brno (*Data Brno*, 2022). The data contain information on noise levels in the city of Brno expressed as average daily decibel levels with confidence interval. Upper level of confidence interval for each ZA was used in further data analysis.

## Socio-economic indicators

### Unemployment rate (UNP)

The unemployment rate was obtained from Population and Housing Census 2011 (CZSO, 2020). The data in city district spatial resolution was transformed in ZAs in the city of Brno. The unemployment data are related only to the economically active portion of population and excludes not working retired people, not working students and apprentices, homemakers, children of preschool age and other dependent people. Considered unemployed were all people who on the day of data collection i) were 15 years of age; ii) had no employment; iii) were economically active. Final data on unemployment were expressed as percent of unemployed in each ZA.

### Education (ED)

The highest education level was obtained from Population and Housing Census 2011 (CZSO, 2020). The data in city district spatial resolution was transformed in ZAs in the city of Brno. The education level was surveyed on people aged 15 years and older, considering only the highest complete level of education. The three levels of education were distinguished: i) elementary education/no education; ii) hight school education; iii) university education. Elementary/no education was further used in the analysis expressed as percent for each ZA.

# Interquartile range of air pollutants

SI Table 3 Pollutant and interquartile range

| **Pollutant** | **Interquartile range (µg/m^3^)** |
| --- | --- |
| PM_2.5_ | 3.36 |
| PM_10_ | 3.77 |
| NO_2_ | 8.92 |
| Benzene | 0.5 |
| SO_2_ | 1.71 |
| PM_2.5_ (5 ya) | 4.23 |
| PM_10_ (5 ya) | 3.13 |
| NO_2_ (5 ya) | 9.68 |
| Benzene (5 ya) | 0.3 |

**5ya** – 5-year average.

# Correlation matrix

SI Table 4 Spearman correlation of health conditions with statistical significance (asterisks).

| **Variable** | **HC1** | **HC2** | **HC3** | **HC4** | **HC5** | **HC6** |
| --- | --- | --- | --- | --- | --- | --- |
| **HC2** | 0.2** |  |  |  |  |  |
| **HC3** | 0.37**** | 0.44**** |  |  |  |  |
| **HC4** | 0.4**** | 0.069 | 0.085 |  |  |  |
| **HC5** | 0.048 | -0.4**** | -0.21** | 0.42**** |  |  |
| **HC6** | 0.0062 | 0.41**** | 0.13* | 0.17** | -0.21** |  |
| **HC7** | -0.17** | 0.14* | 0.043 | -0.047 | 0.12 | 0.32**** |

*****significant p≤0.05; ******significant p≤0.01; *******significant p≤0.001; ********significant p≤0.0001; **HC1** - Prevalence of acute and chronic bronchitis (0-19); **HC2** - Incidence of chronic bronchitis (20+); **HC3** - Incidence of asthma in asthmatic children (0-19); **HC4** - Hospitalization (respiratory diseases, all ages); **HC5** - Hospitalization (respiratory diseases, 65+); **HC6** - CVD hospitalization (including stroke, all ages), **HC7** - CVD hospitalization (including stroke, 65+).

SI Table 5 Spearman correlation of annual AQ, build-up area indicators and socio-economic indicators with statistical significance (asterisks).

| **Variable** | **PM_2.5_** | **PM_10_** | **NO_2_** | **Benzene** | **SO_2_** | **NO_X_** | **DUF** | **CUF** | **ICA** | **RN** | **GS** | **UNP** | **NL** |
| --- | --- | --- | --- | --- | --- | --- | --- | --- | --- | --- | --- | --- | --- |
| **PM_10_** | 0.86**** |  |  |  |  |  |  |  |  |  |  |  |  |
| **NO_2_** | 0.67**** | 0.75**** |  |  |  |  |  |  |  |  |  |  |  |
| **Benzene** | 0.55**** | 0.63**** | 0.61**** |  |  |  |  |  |  |  |  |  |  |
| **SO_2_** | 0.51**** | 0.5**** | 0.48**** | 0.51**** |  |  |  |  |  |  |  |  |  |
| **NO_X_** | 0.5**** | 0.56**** | 0.9**** | 0.48**** | 0.56**** |  |  |  |  |  |  |  |  |
| **DUF** | 0.27**** | 0.27**** | 0.46**** | 0.18** | 0.39**** | 0.51**** |  |  |  |  |  |  |  |
| **CUF** | 0.084 | 0.1 | 0.23*** | 0.11 | 0.11 | 0.24*** | 0.28**** |  |  |  |  |  |  |
| **ICA** | 0.21** | 0.29**** | 0.55**** | 0.29**** | 0.11 | 0.52**** | -0.0055 | 0.13 |  |  |  |  |  |
| **RN** | 0.45**** | 0.5**** | 0.69**** | 0.32**** | 0.44**** | 0.72**** | 0.62**** | 0.09 | 0.28**** |  |  |  |  |
| **GS** | -0.23*** | -0.33**** | -0.5**** | -0.29**** | -0.015 | -0.43**** | 0.11 | -0.31**** | -0.64**** | -0.12 |  |  |  |
| **UNP** | 0.32**** | 0.42**** | 0.62**** | 0.28**** | 0.29**** | 0.62**** | 0.32**** | 0.3**** | 0.49**** | 0.42**** | -0.3**** |  |  |
| **NL** | 0.27**** | 0.34**** | 0.51**** | 0.26**** | 0.14* | 0.48**** | -0.13* | 0.12 | 0.5**** | 0.15* | -0.52**** | 0.27**** |  |
| **ED** | 0.15* | 0.28**** | 0.37**** | 0.2** | -0.037 | 0.31**** | -0.072 | 0.088 | 0.38**** | 0.22*** | -0.45**** | 0.46**** | 0.36**** |

*****significant p≤0.05; ******significant p≤0.01; *******significant p≤0.001; ********significant p≤0.0001; **DUF** – Discontinuous urban fabric; **CUF** – Continuous urban fabric; **ICA** – Industrial and commercial areas; **RN** – Road network; **GS** – Green space; **UNP** –Unemployment rate; **NL** – Noise levels; **ED** – Education.

SI Table 6 Spearman correlation of 5-year mean AQ, build-up area and urban green space indicators with statistical significance (asterisks).

| **Variable** | **PM_2.5_** | **PM_10_** | **NO_2_** | **Benzene** | **B[a]P** | **As** | **Pb** | **Ni** | **Cd** | **DUF** | **CUF** | **ICA** | **RN** | **GS** | **UNP** | **NL** |
| --- | --- | --- | --- | --- | --- | --- | --- | --- | --- | --- | --- | --- | --- | --- | --- | --- |
| **PM_10_** | 0.79**** |  |  |  |  |  |  |  |  |  |  |  |  |  |  |  |
| **NO_2_** | 0.66**** | 0.89**** |  |  |  |  |  |  |  |  |  |  |  |  |  |  |
| **Benzene** | 0.42**** | 0.65**** | 0.72**** |  |  |  |  |  |  |  |  |  |  |  |  |  |
| **B[a]P** | 0.83**** | 0.91**** | 0.73**** | 0.52**** |  |  |  |  |  |  |  |  |  |  |  |  |
| **As** | -0.049 | 0.14* | -0.11 | -0.11 | 0.18** |  |  |  |  |  |  |  |  |  |  |  |
| **Pb** | 0.84**** | 0.91**** | 0.77**** | 0.53**** | 0.89**** | 0.16* |  |  |  |  |  |  |  |  |  |  |
| **Ni** | 0.84**** | 0.93**** | 0.84**** | 0.63**** | 0.93**** | -0.011 | 0.9**** |  |  |  |  |  |  |  |  |  |
| **Cd** | 0.64**** | 0.44**** | 0.2** | -0.028 | 0.64**** | 0.17* | 0.6**** | 0.53**** |  |  |  |  |  |  |  |  |
| **DUF** | 0.3**** | 0.32**** | 0.46**** | 0.38**** | 0.23*** | -0.39**** | 0.28**** | 0.4**** | -0.14* |  |  |  |  |  |  |  |
| **CUF** | 0.093 | 0.14* | 0.21** | 0.25*** | 0.071 | -0.44**** | 0.12 | 0.17* | -0.13 | 0.28**** |  |  |  |  |  |  |
| **ICA** | 0.15* | 0.38**** | 0.55**** | 0.48**** | 0.3**** | -0.2** | 0.29**** | 0.36**** | -0.043 | -0.0055 | 0.13 |  |  |  |  |  |
| **RN** | 0.44**** | 0.61**** | 0.71**** | 0.58**** | 0.44**** | -0.2** | 0.48**** | 0.57**** | -0.083 | 0.62**** | 0.09 | 0.28**** |  |  |  |  |
| **GS** | -0.15* | -0.42**** | -0.49**** | -0.44**** | -0.34**** | -0.0035 | -0.31**** | -0.34**** | 0.013 | 0.11 | -0.31**** | -0.64**** | -0.12 |  |  |  |
| **UNP** | 0.26*** | 0.54**** | 0.65**** | 0.49**** | 0.34**** | -0.18** | 0.45**** | 0.46**** | -0.037 | 0.32**** | 0.3**** | 0.49**** | 0.42**** | -0.3**** |  |  |
| **NL** | 0.2** | 0.44**** | 0.54**** | 0.36**** | 0.33**** | 0.096 | 0.32**** | 0.32**** | 0.02 | -0.13* | 0.12 | 0.5**** | 0.15* | -0.52**** | 0.27**** |  |
| **ED** | 0.059 | 0.38**** | 0.39**** | 0.23*** | 0.27**** | 0.13* | 0.3**** | 0.23*** | 0.053 | -0.072 | 0.088 | 0.38**** | 0.22*** | -0.45**** | 0.46**** | 0.36**** |

*****significant p≤0.05; ******significant p≤0.01; *******significant p≤0.001; ********significant p≤0.0001; **B[a]P** – benzo[a]pyrene; **DUF** – Discontinuous urban fabric; **CUF** – Continuous urban fabric; **ICA** – Industrial and commercial areas; **RN** – Road network; **GS** – Green space; **UNP** –Unemployment rate; **NL** – Noise levels; **ED** – Education.

# Statistically significant GLMs

## Bivariate GLMs

SI Table 7 Statistically significant bivariate GLMs of HCs and AQ indicators expressed as relative risk (RR) with 95% confidence interval (CI), and p values adjusted according to Benjamini and Hochberg (Benjamini and Hochberg, 1995).

| **HC** | **Pollutant** | **RR** | **95% CI** | **Adjusted p value** |
| --- | --- | --- | --- | --- |
| Bronchitis (HC1) | SO_2_ | 0.934 | [0.895 to 0.973] | 0.0015 |
| Bronchitis (HC2) | PM_2.5_ | 1.284 | [1.187 to 1.39] | <0.0001 |
| Bronchitis (HC2) | PM_10_ | 1.429 | [1.265 to 1.615] | <0.0001 |
| Bronchitis (HC2) | NO_2_ | 1.282 | [1.129 to 1.456] | 0.0002 |
| Bronchitis (HC2) | Benzene | 1.759 | [1.597 to 1.937] | <0.0001 |
| Bronchitis (HC2) | PM_2.5_ (5 ya) | 1.297 | [1.192 to 1.412] | <0.0001 |
| Asthma (HC3) | PM_10_ | 1.473 | [1.289 to 1.683] | <0.0001 |
| Asthma (HC3) | Benzene | 1.614 | [1.406 to 1.853] | <0.0001 |
| Asthma (HC3) | SO_2_ | 0.858 | [0.776 to 0.948] | 0.0033 |
| Asthma (HC3) | NO_2_ (5 ya) | 0.875 | [0.815 to 0.94] | 0.0003 |
| Asthma (HC3) | Benzene (5 ya) | 0.908 | [0.859 to 0.961] | 0.001 |
| Resp. hosp. (HC4) | NO_2_ (5 ya) | 1.044 | [1.003 to 1.088] | 0.0442 |
| Resp. hosp. (HC4) | Benzene (5 ya) | 1.041 | [1.01 to 1.073] | 0.0115 |
| Resp. hosp. (HC5) | PM_2.5_ | 0.911 | [0.856 to 0.969] | 0.0042 |
| Resp. hosp. (HC5) | Benzene | 0.887 | [0.832 to 0.947] | 0.0004 |
| Resp. hosp. (HC5) | PM_2.5_ (5 ya) | 0.896 | [0.839 to 0.957] | 0.0014 |
| Resp. hosp. (HC5) | PM_10_ (5 ya) | 0.938 | [0.881 to 0.997] | 0.0483 |
| CVD hosp. (HC6) | PM_2.5_ | 1.08 | [1.043 to 1.118] | <0.0001 |
| CVD hosp. (HC6) | PM_10_ | 1.086 | [1.048 to 1.126] | <0.0001 |
| CVD hosp. (HC6) | NO_2_ | 1.146 | [1.104 to 1.19] | <0.0001 |
| CVD hosp. (HC6) | Benzene | 1.133 | [1.097 to 1.17] | <0.0001 |
| CVD hosp. (HC6) | SO_2_ | 1.101 | [1.061 to 1.143] | <0.0001 |
| CVD hosp. (HC6) | PM_2.5_ (5 ya) | 1.1 | [1.062 to 1.14] | <0.0001 |
| CVD hosp. (HC6) | PM_10_ (5 ya) | 1.115 | [1.074 to 1.157] | <0.0001 |
| CVD hosp. (HC6) | NO_2_ (5 ya) | 1.162 | [1.113 to 1.213] | <0.0001 |
| CVD hosp. (HC6) | Benzene (5 ya) | 1.124 | [1.088 to 1.161] | <0.0001 |
| CVD hosp. (HC7) | PM_2.5_ | 1.032 | [1.002 to 1.063] | 0.0476 |
| CVD hosp. (HC7) | PM_10_ | 1.051 | [1.022 to 1.082] | 0.0006 |
| CVD hosp. (HC7) | NO_2_ | 1.098 | [1.065 to 1.132] | <0.0001 |
| CVD hosp. (HC7) | Benzene | 1.085 | [1.053 to 1.117] | <0.0001 |
| CVD hosp. (HC7) | SO_2_ | 1.065 | [1.031 to 1.1] | 0.0002 |
| CVD hosp. (HC7) | PM_2.5_ (5 ya) | 1.047 | [1.017 to 1.078] | 0.0026 |
| CVD hosp. (HC7) | PM_10_ (5 ya) | 1.093 | [1.06 to 1.128] | <0.0001 |
| CVD hosp. (HC7) | NO_2_ (5 ya) | 1.12 | [1.079 to 1.163] | <0.0001 |
| CVD hosp. (HC7) | Benzene (5 ya) | 1.075 | [1.038 to 1.113] | 0.0001 |

**HC1** - Prevalence of acute and chronic bronchitis (0-19); **HC2** - Incidence of chronic bronchitis (20+); **HC3** - Incidence of asthma in asthmatic children (0-19); **HC4** - Hospitalization (respiratory diseases, all ages); **HC5** - Hospitalization (respiratory diseases, 65+); **HC6** - CVD hospitalization (including stroke, all ages), **HC7** - CVD hospitalization (including stroke, 65+); **5ya** – 5-year average.

## Multivariate GLMs adjusted for BA and SC

SI Table 8 Statistically significant multivariate GLMs of HCs, AQ, BA and SC indicators expressed as relative risk (RR) with 95% confidence interval (CI), and p values adjusted according to Benjamini and Hochberg (Benjamini and Hochberg, 1995).

| **HC** | **Pollutant** | **RR** | **95% CI** | **Adjusted p value** |
| --- | --- | --- | --- | --- |
| Bronchitis (HC2) | PM_2.5_ | 1.355 | [1.249 to 1.471] | <0.0001 |
| Bronchitis (HC2) | PM_10_ | 1.243 | [1.101 to 1.403] | 0.0018 |
| Bronchitis (HC2) | NO_2_ | 1.483 | [1.227 to 1.792] | 0.0002 |
| Bronchitis (HC2) | Benzene | 1.552 | [1.415 to 1.704] | <0.0001 |
| Bronchitis (HC2) | SO_2_ | 1.18 | [1.061 to 1.313] | 0.0072 |
| Bronchitis (HC2) | PM_2.5_ (5 ya) | 1.347 | [1.236 to 1.468] | <0.0001 |
| Bronchitis (HC2) | PM_10_ (5 ya) | 1.228 | [1.082 to 1.394] | 0.005 |
| Asthma (HC3) | PM_2.5_ | 1.115 | [1.058 to 1.175] | 0.0002 |
| Asthma (HC3) | Benzene | 1.108 | [1.036 to 1.184] | 0.0077 |
| Asthma (HC3) | PM_2.5_ (5 ya) | 1.128 | [1.073 to 1.185] | <0.0001 |
| Asthma (HC3) | PM_10_ (5 ya) | 1.171 | [1.083 to 1.266] | 0.0004 |
| Asthma (HC3) | NO_2_ (5 ya) | 1.346 | [1.152 to 1.573] | 0.0008 |
| Resp. hosp. (HC5) | PM_2.5_ | 0.862 | [0.807 to 0.921] | <0.0001 |
| Resp. hosp. (HC5) | PM_10_ | 0.862 | [0.795 to 0.935] | 0.0015 |
| Resp. hosp. (HC5) | NO_2_ | 0.737 | [0.655 to 0.829] | <0.0001 |
| Resp. hosp. (HC5) | Benzene | 0.849 | [0.787 to 0.916] | <0.0001 |
| Resp. hosp. (HC5) | SO_2_ | 0.896 | [0.841 to 0.955] | 0.0026 |
| Resp. hosp. (HC5) | PM_2.5_ (5 ya) | 0.862 | [0.803 to 0.926] | 0.0002 |
| Resp. hosp. (HC5) | PM_10_ (5 ya) | 0.845 | [0.775 to 0.922] | 0.0007 |
| Resp. hosp. (HC5) | NO_2_ (5 ya) | 0.698 | [0.596 to 0.819] | <0.0001 |
| CVD hosp. (HC6) | NO_2_ | 1.16 | [1.075 to 1.251] | 0.0006 |
| CVD hosp. (HC6) | Benzene | 1.102 | [1.062 to 1.143] | <0.0001 |
| CVD hosp. (HC6) | SO_2_ | 1.068 | [1.029 to 1.108] | 0.0018 |
| CVD hosp. (HC6) | PM_2.5_ (5 ya) | 1.061 | [1.029 to 1.094] | 0.0007 |
| CVD hosp. (HC6) | PM_10_ (5 ya) | 1.089 | [1.045 to 1.135] | 0.0003 |
| CVD hosp. (HC6) | NO_2_ (5 ya) | 1.218 | [1.119 to 1.325] | <0.0001 |
| CVD hosp. (HC6) | Benzene (5 ya) | 1.082 | [1.039 to 1.126] | 0.0006 |
| CVD hosp. (HC7) | Benzene | 1.06 | [1.023 to 1.098] | 0.0045 |
| CVD hosp. (HC7) | SO_2_ | 1.054 | [1.019 to 1.091] | 0.0076 |

**HC2** - Incidence of chronic bronchitis (20+); **HC3** - Incidence of asthma in asthmatic children (0-19); **HC5** - Hospitalization (respiratory diseases, 65+); **HC6** - CVD hospitalization (including stroke, all ages), **HC7** - CVD hospitalization (including stroke, 65+); **5ya** – 5-year average.

# Long-term trends in AQ in the Czech Republic counties

**SI Table 9** to **SI Table 17** shows 3 of the most polluted and 3 of the least polluted counties (1-77) in the Czech Republic by long-term mean concentration (5-year mean) and position of the city of Brno. Counties are listed in ascending order (1 least polluted, 77 most polluted). Order of the city of Brno (Brno-mesto) is shown for the pollutants where the city of Brno is not on the 1-3^rd^ or 75-77^th^ place. Additionally. Prague (Praha) is technically not a county. but the country region. However, we consider Prague as a county for this purpose. And the city of Brno (Brno-mesto) and the city of Ostrava (Ostrava-mesto) are in fact cities. but also regarded as a county.

SI Table 9 PM_2.5_ levels (µg/m^3^ ,2007-2001 to 2014-2018) in counties in the Czech Republic.

| **Order** | **County** | **Mean** | **SD** | **Median** | **Kendall correlation** |
| --- | --- | --- | --- | --- | --- |
| 1 | Prachatice | 9.62 | 0.732 | 9.75 | -0.57* |
| 2 | Český Krumlov | 10.05 | 0.424 | 10.08 | -0.64* |
| 3 | Karlovy Vary | 10.72 | 0.327 | 10.73 | 0.07 |
| … | … | … | … | … | … |
| **73** | **Brno-město** | **22** | **3.199** | **21.41** | **-0.93**** |
| … | … | … | … | … | … |
| 75 | Nový Jičín | 22.62 | 1.089 | 22.54 | -0.43 |
| 76 | Ostrava-město | 29.64 | 2.59 | 30.41 | -0.71* |
| 77 | Karviná | 32.88 | 2.715 | 33.44 | -0.64* |

*****significant p≤0.05; ******significant p≤0.01; *******significant p≤0.001; ********significant p≤0.0001.

SI Table 10 PM_10_ levels (µg/m^3^ ,2007-2001 to 2014-2018) in counties in the Czech Republic.

| **Order** | **County** | **Mean** | **SD** | **Median** | **Kendall correlation** |
| --- | --- | --- | --- | --- | --- |
| 1 | Prachatice | 10.98 | 0.498 | 11.09 | 0.71* |
| 2 | Český Krumlov | 12.02 | 0.643 | 12.17 | 0.79** |
| 3 | Klatovy | 13.67 | 0.364 | 13.87 | 0.36 |
| … | … | … | … | … | … |
| **71** | **Brno-město** | **26.6** | **2.321** | **26.66** | **-0.86**** |
| … | … | … | … | … | … |
| 75 | Nový Jičín | 29.29 | 1.771 | 29.94 | -0.64* |
| 76 | Ostrava-město | 37.9 | 3.329 | 39.01 | -0.64* |
| 77 | Karviná | 42.55 | 3.788 | 43.72 | -0.71* |

*****significant p≤0.05; ******significant p≤0.01; *******significant p≤0.001; ********significant p≤0.0001.

SI Table 11 NO_2_ levels (µg/m^3^,2007-2001 to 2014-2018) in counties in the Czech Republic.

| **Order** | **County** | **Mean** | **SD** | **Median** | **Kendall correlation** |
| --- | --- | --- | --- | --- | --- |
| 1 | Prachatice | 6.93 | 0.758 | 7.01 | -0.64* |
| 2 | Český Krumlov | 7.01 | 0.521 | 6.94 | -0.21 |
| 3 | Klatovy | 7.95 | 0.794 | 7.97 | -0.5 |
| … | … | … | … | … | … |
| 75 | Ostrava-město | 18.53 | 1.196 | 18.47 | -0.64* |
| **76** | **Brno-město** | **21.99** | **5.971** | **21.47** | **-1***** |
| 77 | Praha | 22.9 | 2.812 | 23.11 | -1*** |

*****significant p≤0.05; ******significant p≤0.01; *******significant p≤0.001; ********significant p≤0.0001.

SI Table 12 Benzene levels (µg/m^3^,2007-2001 to 2014-2018) in counties in the Czech Republic.

| **Order** | **County** | **Mean** | **SD** | **Median** | **Kendall correlation** |
| --- | --- | --- | --- | --- | --- |
| 1 | Prachatice | 0.58 | 0.087 | 0.56 | -0.29ns |
| 2 | Český Krumlov | 0.61 | 0.063 | 0.62 | 0 |
| 3 | Klatovy | 0.67 | 0.101 | 0.69 | 0 |
| … | … | … | … | … | … |
| **71** | **Brno-město** | **1.5** | **0.243** | **1.53** | **0** |
| … | … | … | … | … | … |
| 75 | Nový Jičín | 1.75 | 0.19 | 1.79 | -0.71* |
| 76 | Karviná | 2.42 | 0.355 | 2.36 | -1*** |
| 77 | Ostrava-město | 2.59 | 0.397 | 2.64 | -1*** |

*****significant p≤0.05; ******significant p≤0.01; *******significant p≤0.001; ********significant p≤0.0001.

SI Table 13 Benzo(a)pyrene levels (ng/m^3^ ,2007-2001 to 2014-2018) in counties in the Czech Republic.

| **Order** | **County** | **Mean** | **SD** | **Median** | **Kendall correlation** |
| --- | --- | --- | --- | --- | --- |
| 1 | Prachatice | 0.19 | 0.05 | 0.19 | -0.71* |
| 2 | Český Krumlov | 0.2 | 0.07 | 0.2 | -0.71* |
| 3 | Cheb | 0.25 | 0.06 | 0.24 | -0.57* |
| … | … | … | … | … | … |
| **60** | **Brno-město** | **0.9** | **0.18** | **0.84** | **-0.86**** |
| … | … | … | … | … | … |
| 75 | Frýdek-Místek | 1.74 | 0.22 | 1.84 | 0.64* |
| 76 | Karviná | 3.57 | 0.28 | 3.54 | 0.36 |
| 77 | Ostrava-město | 3.72 | 0.63 | 3.86 | -0.93** |

*****significant p≤0.05; ******significant p≤0.01; *******significant p≤0.001; ********significant p≤0.0001.

SI Table 14 Arsenic levels (ng/m^3^ ,2007-2001 to 2014-2018) in counties in the Czech Republic.

| **Order** | **County** | **Mean** | **SD** | **Median** | **Kendall correlation** |
| --- | --- | --- | --- | --- | --- |
| 1 | Český Krumlov | 0.85 | 0.23 | 0.97 | -1*** |
| 2 | Prachatice | 0.87 | 0.23 | 0.99 | -0.5ns |
| 3 | Třebíč | 0.89 | 0.1 | 0.93 | -0.64* |
| … | … | … | … | … | … |
| **7** | **Brno-město** | **0.92** | **0.1** | **0.93** | **0.07** |
| … | … | … | … | … | … |
| 75 | Praha | 2.07 | 0.14 | 2.09 | -0.86** |
| 76 | Ostrava-město | 2.08 | 0.36 | 1.99 | -0.86** |
| 77 | Kladno | 2.4 | 0.35 | 2.42 | -1*** |

*****significant p≤0.05; ******significant p≤0.01; *******significant p≤0.001; ********significant p≤0.0001.

SI Table 15 Lead levels (ng/m^3^ ,2007-2001 to 2014-2018) in counties in the Czech Republic.

| **Order** | **County** | **Mean** | **SD** | **Median** | **Kendall correlation** |
| --- | --- | --- | --- | --- | --- |
| 1 | Český Krumlov | 2.55 | 1.02 | 2.35 | -0.86** |
| 2 | Prachatice | 2.66 | 1.11 | 2.39 | -0.79** |
| 3 | Jindřichův Hradec | 3.62 | 1.29 | 3.39 | -0.93** |
| … | … | … | … | … | … |
| **68** | **Brno-město** | **8.1** | **1.55** | **8.08** | **-1***** |
| … | … | … | … | … | … |
| 75 | Frýdek-Místek | 11.48 | 1.19 | 11.55 | -0.93** |
| 76 | Ostrava-město | 17.23 | 2.94 | 18.65 | -0.86** |
| 77 | Karviná | 18.14 | 3.35 | 19.8 | -0.64* |

*****significant p≤0.05; ******significant p≤0.01; *******significant p≤0.001; ********significant p≤0.0001.

SI Table 16 Nickel levels (ng/m3,2007-2001 to 2014-2018) in counties in the Czech Republic.

| **Order** | **County** | **Mean** | **SD** | **Median** | **Kendall correlation** |
| --- | --- | --- | --- | --- | --- |
| 1 | Trutnov | 0.67 | 0.17 | 0.7 | -1*** |
| 2 | Jeseník | 0.68 | 0.08 | 0.7 | -0.57* |
| 3 | Bruntál | 0.7 | 0.09 | 0.71 | -0.57* |
| … | … | … | … | … | … |
| 75 | Karviná | 1.43 | 0.24 | 1.45 | 0.14 |
| 76 | Plzeň-město | 1.47 | 0.61 | 1.65 | -1*** |
| **77** | **Brno-město** | **1.72** | **0.54** | **1.64** | **-0.93**** |

*****significant p≤0.05; ******significant p≤0.01; *******significant p≤0.001; ********significant p≤0.0001.

SI Table 17 Cadmium levels (ng/m3 ,2007-2001 to 2014-2018) in counties in the Czech Republic.

| **Order** | **County** | **Mean** | **SD** | **Median** | **Kendall correlation** |
| --- | --- | --- | --- | --- | --- |
| 1 | Znojmo | 0.257 | 0.049 | 0.266 | -1*** |
| **2** | **Brno-město** | **0.258** | **0.054** | **0.248** | **-1***** |
| 3 | Prachatice | 0.263 | 0.058 | 0.287 | -0.64* |
| … | … | … | … | … | … |
| 75 | Ostrava-město | 0.813 | 0.172 | 0.852 | -0.79** |
| 76 | Semily | 0.872 | 0.1 | 0.878 | 0.07 |
| 77 | Jablonec nad Nisou | 1.406 | 0.212 | 1.464 | 0.5 |

*****significant p≤0.05; ******significant p≤0.01; *******significant p≤0.001; ********significant p≤0.0001.

# The distribution of elderly people (65+) within the city of Brno


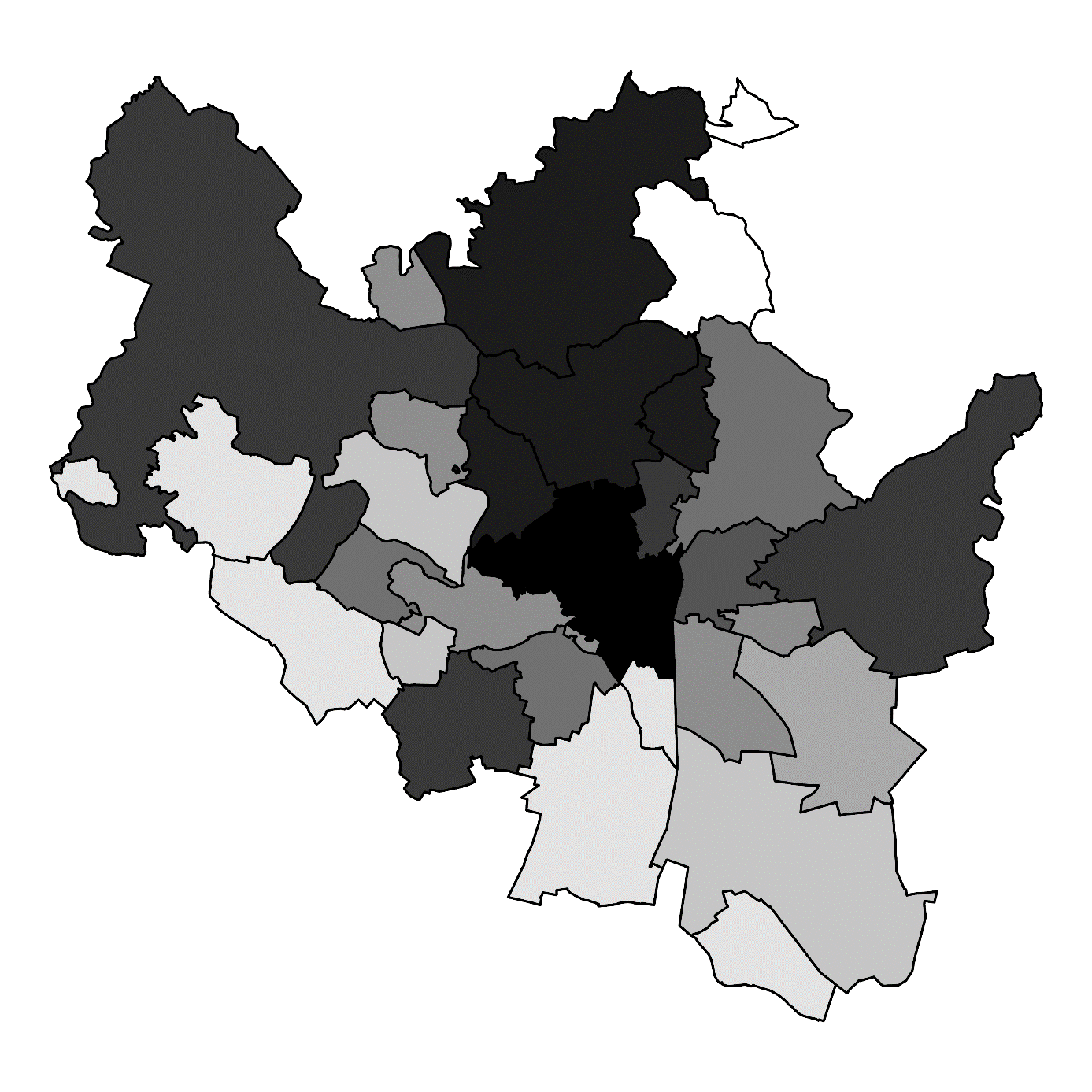


SI Figure 4 The distribution of elderly people (65+) within the city of Brno, darker the colour, higher the number of people aged 65+.

# References

Benjamini, Y. and Hochberg, Y. (1995) ‘Controlling the False Discovery Rate: A Practical and Powerful Approach to Multiple Testing’, *Journal of the Royal Statistical Society*, 57(1), pp. 289–300. Available at: https://www.jstor.org/stable/2346101.

CLC (2018) *CORINE Land Cover*. Available at: https://land.copernicus.eu/pan-european/corine-land-cover/clc2018 (Accessed: 12 April 2021).

COSMC (2021) *Digital geographical model of territory of the Czech Republic (Data50) - Vegetation and surface*. Available at: https://geoportal.cuzk.cz/(S(1ywgh1b2lc2mcgijfekgr4gk))/Default.aspx?menu=22906&mode=TextMeta&side=mapy_data50&metadataID=CZ-CUZK-DATA50-VEGETACE_POVRCH-V.

CZSO (2020) *Population and housing census*. Available at: https://www.czso.cz/csu/czso/population-and-housing-census (Accessed: 1 August 2020).

*Data Brno* (2022). Available at: https://datahub.brno.cz/search?collection=Dataset (Accessed: 18 July 2022).

Florczyk, A. J. *et al.* (2019) *GHSL data package 2019*, *Publications Office of the European Union*. doi: 10.2760/0726.

GeoFabrik (2016) *Geofabrik: Download server for openstreetmap data*. Available at: https://download.geofabrik.de/.

Kosztra, B. *et al.* (2017) *Updated CLC illustrated nomenclature guidelines*, *Final Report by European Environmental Agency*. Vien. Available at: https://land.copernicus.eu/user-corner/technical-library/corine-land-cover-nomenclature-guidelines/docs/pdf/CLC2018_Nomenclature_illustrated_guide_20190510.pdf.

OSM (2022) *OpenStreetMap contributors*. Available at: https://www.openstreetmap.org/#map=12/49.1954/16.6112.
